# In This Issue

**DOI:** 10.1002/cfg.166

**Published:** 2002-04


**Comparative and Functional Genomics**
*Comp Funct Genom* 2002; **3**: 89–90.
Published online 12 March 2002 in Wiley InterScience (www.interscience.wiley.com). **DOI:** 10.1002/cfg.166

### In this issue

### A web-based relational database for microarray data

Madhusmita Mitra and colleagues report on the design and development of StressDB, a locally installable, web-based, relational microarray database aimed at small communities of cDNA microarray technology users. StressDB allows users to manage microarray data and data-related biological information over the Internet using a web browser.

### A European pathogenic microorganism proteome database

Klaus-Peter Pleißner *et al.* present a 2D-PAGE database with a relational database structure based on MS-Access and MySQL. The system can be used to compare data with that already in the database, or to construct a new proteome database, by users with little bioinformatics experience.

### Reviews from the ESF 'Integrated Approaches For Functional Genomics' Programme Workshop 'Integrating Data In Genomics And Proteomics'

Javier Paz-Ares describes REGIA, the REgulatory Gene Initiative in *Arabidopsis*, in which 29 European laboratories are using a combination of approaches to determine the function of virtually all transcription factors in this model plant.

Ramon Allonso-Allende and colleagues report on the challenges faced, and the progress made so far, by the REGIA bioinformatics workpackage members in developing RegiaDB, a relational database for the project.

### The Biopathways Consortium

Eric Neumann and Vincent Schachter present the progress made by the BioPathways Consortium (BPC) since its formation in June 2000. The consortium has a dual mission: to propose and advance post-genomic informatics, and to encourage the adoption of practical guidelines and standards.

### Do online bioinformatics searches affect the confidentiality of novel sequences?

A key requirement for patenting a sequence is novelty, and it is also necessary to have evidence of a potential use for it. However, on-line sequence comparisons require the sequence to be sent out as a query term, potentially destroying its novelty. Anton Hutter's review addresses key areas for concern and suggests ways to reduce the risk involved.

### Special Section of selected reviews from the Plant, Animal and Microbe Genomes X conference

### Conference reviews

**Mary Polacco** and colleagues introduce MaizeDB as a functional genomics tool, and describe new ways to access the data. Their annotation of the genetic maps and related objects incorporates information on gene products, and phenotypic and agronomic trait data all of which relate to function.

**Pankaj Jaiswal *et al.*** present Gramene, a comparative database for cereal crops and a community resource for rice. The Gramene team are integrating Gene Ontology terms into rice gene annotation, and have made progress in developing a Trait Ontology as part of the Plant Ontology Consortium.

**The Plant Ontology Consortium** draws members from the International Rice Research Institute, The *Arabidopsis* Information Resource, MaizeDB and Gramene. The goal of the Consortium is to produce structured controlled vocabularies, arranged in ontologies, which can be applied to plant-based database information.

**Richard Smith** describes a new method for mass spectrometric analysis of proteomes. This approach combines high resolution capillary liquid chromatography separations and Fourier transform ion cyclotron resonance (FTICR) mass spectrometry,

Copyright © 2002 John Wiley & Sons, Ltd.

and has so far been tested on *Deinococcus radiodurans* and *Saccharomyces cerevisiae*.

**Ben Trevaskis** and colleagues review recent advances in the study of plant cell differentiation during nodule formation in leguminous plants, highlighting the role of transcriptomic, proteomic and metabolomic analyses.

**Xin Li and Yuelin Zhang** present the DELETEAGENE strategy for plant mutagenesis, which has been tested on *Arabidopsis*. In this deletion-based gene knockout system, random deletion libraries are created by fast neutron mutagenesis and screened by PCR using primers flanking the target gene.

**Yongbiao Xue and Zhihong Xu** provide an overview of the Chinese rice functional genomics program. Twenty Chinese research groups are participating in the program, which aims to identify genes related to flowering, plant architecture, fertility, reproduction, metabolic control and stress responses in rice.

**Shanna Moore** and colleagues have produced a 9200 element tomato microarray that will soon be made available to the public. They have used the array to study fruit ripening and development and have shown that the array can be used for other *Solanacae*, such as peppers and potatoes.

**Wusirika Ramakrishna *et al*.** have sequenced BAC clones covering the *Rp1* disease resistance locus and the *Waxy1* (*Wx1*) locus in a range of grasses and made a comparative study of these regions. Here they review their findings and compare them to those observed in other regions.

**Clemens Suter-Crazzolara and Günther Kurapkat** provide a brief overview of the use of ArraySCOUT™ by the *Listeria* sequencing consortium to manage the data produced in the recent comparison of *Listeria monocytogenes* with *Listeria innocua*.

**Damian Gessler** presents ISYS, a system for integrating varied bioinformatic tools and online resources, rather than the data they hold. The system enables the researcher to use multiple tools at once, with background tools responding to selections made in the active tool.

**Richard Bruskiewich** provides a brief summary of the recommendations for plant bioinformatics that were discussed at the recent NSF and NPGI satellites at PAMGX.

### Announcement

SPRIG (Specialized Plant Resources for Informatics and Genomics) is an initiative designed to bring together plant bioinformaticians, to facilitate the exchange of information, protocols and resources and avoid too much 'reinvention of the wheel'.

### Meeting Highlights

To complement our selected reviews, we bring you a detailed report from PAMGX, covering the plenary sessions, and workshops on comparative genomics, functional genomics, databases, gene systematics and nomenclature, ontology for databases, bioinformatics, the international grass genome initiative, rice, *Arabidopsis* and microbial functional genomics.

### Meeting Review: Reproductive Genomics

Gerard Gibbs and Moira O'Bryan report on this joint Prince Henry's Institute of Medical Research and Monash Institute for Reproduction and Development symposium, held in Melbourne, Australia, in December.

### Featured Organism: *Schizosaccharomyces pombe*

On February 21st the genome sequence of the fission yeast, *Schizosaccharomyces pombe* was published in Nature. We present an in-depth profile of this important model Eukaryote, with comments from Paul Nurse, Iain Hagan, Jurg Bahler, Susan Forsburg, Ramsay McFarlane and Valerie Wood.

Copyright © 2002 John Wiley & Sons, Ltd.

